# Mental Health Surveillance at federal state level – Reporting on psychiatry in Bavaria

**DOI:** 10.25646/8859

**Published:** 2021-12-08

**Authors:** Daniela Blank, Rebekka Redel, Martin Tauscher, Joseph Kuhn

**Affiliations:** 1 Bavarian Health and Food Safety Authority; 2 Association of Statutory Health Insurance Physicians in Bavaria

**Keywords:** MENTAL HEALTH, REPORTING ON PSYCHIATRY, HEALTH REPORTING, INDICATORS, BAVARIA

## Abstract

In Germany, mental health reporting is organised at the federal, federal state and municipal level. At federal level, a number of concepts and approaches are implemented. In 2020 and 2021, in accordance with Article 4 of the Mental Health Assistance Act the first Bavarian Psychiatry Report was prepared. Important data bases include the billing and care data of Bavaria’s Association of Statutory Health Insurance Physicians and the administrative data of the Bavarian districts. The aim is to enhance coordination between these federal state projects and Mental Health Surveillance at national level, in particular regarding the use of health care data.

## 1. Introduction

Health reporting as a data-based form of describing population health is a fundamental public health task and part of the ten core areas of public health (Essential Public Health Operations, EPHO), as formulated by the World Health Organization’s European Regional Office in 2012 [1, 2]. In a pluralistic health care system, health reporting as a basis for planning as well as for mediating cooperation and communication plays a decisive role. This is especially true for the field of mental health with its highly heterogeneous and segmented care structures [3].

In federal health reporting, data on mental health and the provision of mental healthcare is provided primarily by the Robert Koch Institute (RKI) in co-operation with the Federal Statistical Office, as well as by health insurance fund health reports. It is also based on specific modules of nationwide health surveys such as the additional mental health module of the German Health Interview and Examination Survey for Adults (DEGS1-MH) [4] and the survey on mental well-being and behaviour (BELLA), within the framework of the German Health Interview and Examination Survey for Children and Adolescents (KiGGS) [5]. An expansion is currently taking place through the establishment of the national Mental Health Surveillance at the RKI (see Focus article Establishment of a Mental Health Surveillance in Germany: Development of a framework concept and indicator set in this issue of the Journal of Health Monitoring).

Psychiatric care planning in Germany, as corresponding reporting is organised essentially at the specific federal state level. This includes the AOLG (AG Psychiatrie der Obersten Landesgesundheitsbehörden) reports, the most recent one being the 2017 report [6]. No standards for the labelling of federal and municipal level reporting have been established. There are health reports covering mental health issues and psychiatric care plans, which are often standalone and prepared as specialised planning outside of health reporting, but to all intents and purposes they are in effect health reports, and, more recently, also occasionally psychiatry reports.

The choice of topics, data and formats is extremely heterogeneous. The range of topics spans from the description of the epidemiological situation and the number of involuntary admissions to the documentation of prevention and care services in individual service areas, such as medical care, addiction counselling or integration assistance in line with Germany’s social code IX (SGB IX). In some cases, specific disease patterns are examined (e.g. depression [7, 8]) or the focus is on specific settings (e.g. the work environment [9]). In terms of content, these reports draw on data from nationwide RKI health surveys, international studies, official statistics, billing and health care data; in some cases, data from own surveys are presented (e.g. surveys by experts [10, 11]). They also differ in format. For example, in Saxony and Bavaria, the Mental Health Assistance Act (PsychKHG) contains legal provisions regarding reporting cycles. In view of the broad range of topics, data and reporting formats, it is as hard to talk about ‘psychiatric reporting’ in general terms as it is to talk about ‘health reporting’ [12]. This article presents the approach taken in Bavaria’s first psychiatry report, and illustrates the importance of routine healthcare data, which has tended, so far, to play a more minor role in health reporting. This is intended to contribute to the discussion of the on-going design of mental health surveillance in Germany in consideration of the interactions between the regional, federal state and federal levels.

## 2. Thematic focuses and data within Bavaria’s psychiatry report

In 2018, the federal state parliament of Bavaria passed Bavaria’s Mental Health Assistance Act (BayPsychKHG). Article 4 establishes comprehensive psychiatric reporting regarding epidemiology and care. This is the first time that a German federal state government has established regular (triennial) reporting to parliament on mental health in a federal state PsychKHG. The illustration of basic epidemiological data on mental health, disease-related protection and risk factors and available services is meant to further develop prevention and care. This also includes data on services uptake and costs relative to Bavaria’s resident population. The dimensions of the RKI’s federal Mental Health Surveillance format provided the basis to prioritise issues. In the run up to this, mental health was a recurrent topic in Bavarian health reporting [8, 13] – providing a basis psychiatry reporting.

The first report was prepared in 2020/2021. In addition to official statistics and social insurance fund data (pension insurance, health insurance fund reports, etc.), the billing and care data of Bavaria’s Association of Statutory Health Insurance Physicians (KVB) and the Bavarian districts (as providers of supra-local social assistance) are a central data source for the report. KVB data show the diagnoses documented by physicians and psychotherapists in practices for patients covered by statutory health insurance in Bavaria. They allow statements on the diagnosed prevalence at district level and on treatment uptake. The Bavarian districts then provide important structural and process data from complementary care, such as on the availability and use of shared housing places or workshops for people who have mental disabilities. The facts behind an integrative and systematic approach speak for themselves. For example, integration assistance services, as well as basic social services have an impact on the medical-psychotherapeutic care system, insofar as they decisively influence the uptake of services. They do this, for example, by pointing out the available services to patients, encouraging uptake or making treatment needs visible in advance. A good example of this is the crisis services which are available across Bavaria. At the same time, transition rates, for example from specially protected work contexts to the general labour market, are a strong indicator of how well or poorly the system is capable of improving the social participation of people with a long-term mental health condition.

Although the routine data of the KVB have the advantage that they are not distorted by non-participation (as is the case with health surveys) and that they can be updated regularly without much effort, it is important to remember that the frequency of diagnoses is not the same as the frequency of an illness: those who are ill but do not seek medical care do not appear in health care system diagnostic data. Moreover, in many cases physicians are careful to immediately diagnose an illness from the group of mental disorders (International Statistical Classification of Diseases and Related Health Problems, 10th revision; ICD-10: F00–F99). To avoid unnecessary stigmatisation, diagnoses in suspected cases are deliberately assigned with caution and restraint. In addition, the data of the Association of Statutory Health Insurance Physicians of Bavaria do not include the privately insured, who make up about ten per cent of the population.

Between the first quarter of 2019 right up to and including the fourth quarter of 2019, just over 2.8 million patients of all age groups covered by statutory health insurance in Bavaria were diagnosed with a mental disorder (ICD-10: F00–F99) in at least two quarters. In the under-18 age group, there was a total of slightly more than 285,000 children and adolescents. Extrapolated to all people covered by statutory health insurance (28.6% of adults, 16.0% of adolescents), these data correspond to the figures reported by the DEGS1-MH module or the BELLA study. Apart from the different timeframes used, it is important to highlight that the two data sets are not completely congruent. For example, a considerable proportion of people diagnosed with a mental disorder in the DEGS1-MH module (2009– 2012) are not currently in treatment and, conversely, people with severe mental disorders are underrepresented in the health surveys [14].

**Figure 1:**
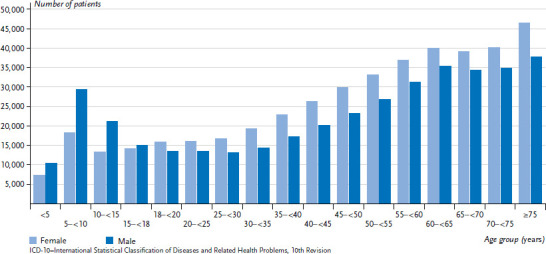
Number of patients with a mental disorder (outpatient diagnoses, ICD-10: F00–F99) per 100,000 patients in Bavaria 2019 by sex and age Source: 2019 billing data form Bavaria’s Association of Statutory Health Insurance Physicians

These discrepancies are particularly evident when comparing age groups. For example, according to KiGGS (BELLA Wave 3, 2009–2012), the frequency of mental health symptoms or disorders in childhood and adolescence is highest among 11- to 13-year-olds compared to the other age groups [15]. However, the billing data of the Association of Statutory Health Insurance Physicians of Bavaria (Figure 1), just like Germany-wide data on health care provision [16], show that ICD-10 F-diagnoses are most frequent in the 5- to 10-year-old age group. This can be attributed to the developmental disorders detected with the start of school, which are documented as F-diagnoses, but which are not actually mental disorders. The German Health Interview and Examination Survey for Children and Adolescents does not systematically record development disorders of speech and language or of the basic skills required for school [13].

The psychiatry report provides a synopsis of diverse sources of data. In terms of content, each of these data sources has its strengths and limitations. While routine data does not provide answers to all questions – as it depends on actual uptake of healthcare – it does show the degree to which mental disorders are represented in the health care system. Despite the fact that the incidence remains essentially stable, this figure has increased over the last two decades. The level of care for people with a mental illness has therefore improved. However, health care provision data also show great differences, for example depending on age or region.

Overall, the data situation on mental health is still patchy in many areas; especially with regard to particularly difficult phases in life, the quality of life of people with mental illness and the economic and social situation of patients with chronic mental health issues. Data on the quality of services and on the forms of co-ordination and co-operation between actors is also limited. For specific care services, such as supported employment (specific support for patients to find work), occupational therapy and sociotherapy, no data are available at all. By expanding the reporting systems at the federal state and federal levels these gaps in the data will need to be closed in the future.

Due to the COVID-19 pandemic, preparing the Bavarian psychiatry report has been considerably more difficult, with only a limited number of external experts taking part in the initial preparation of the report. For the next Bavarian psychiatry report (2024), more external expertise is to be consulted – especially with regard to the scope of data used. A co-operation with federal Mental Health Surveillance at the RKI is planned.

## Key statements

- Healthcare routine data is an important element of mental health surveillance.
- The data complement findings from scientific surveys – not only regarding the provision of care, but also in epidemiology.
- In 2019, just over 2.8 million patients covered by statutory health insurance in Bavaria were diagnosed with mental health issues.
- The prevalence of diagnoses from the group of mental disorders increases steadily in outpatient care from younger adulthood. Men are more affected during childhood and adolescence, whereas women are more affected during adolescence.
